# Spatiotemporal strategies to identify aggressive biology in precancerous breast biopsies

**DOI:** 10.1002/wsbm.1506

**Published:** 2020-10-01

**Authors:** David E. Frankhauser, Tijana Jovanovic-Talisman, Lily Lai, Lisa D. Yee, Lihong V. Wang, Ashish Mahabal, Joseph Geradts, Russell C. Rockne, Jerneja Tomsic, Veronica Jones, Christopher Sistrunk, Gustavo Miranda-Carboni, Eric C. Dietze, Loretta Erhunmwunsee, Terry Hyslop, Victoria L. Seewaldt

**Affiliations:** 1 Department of Population Sciences, City of Hope Comprehensive Cancer Center, Duarte, California; 2 Department of Medical Engineering, California Institute of Technology, Pasadena, California; 3 Center for Data Driven Discovery, California Institute of Technology, Pasadena, California; 4 Department of Pathology, Duke University, Durham, North Carolina; 5 Department of Hematology and Oncology, University of Tennessee, Memphis, Memphis, Tennessee; 6 Department of Biostatistics, Duke University, Durham, North Carolina

**Keywords:** breast imaging, early detection, multiscale models

## Abstract

Over 90% of breast cancer is cured; yet there remain highly aggressive breast cancers that develop rapidly and are extremely difficult to treat, much less prevent. Breast cancers that rapidly develop between breast image screening are called “interval cancers.” The efforts of our team focus on identifying multiscale integrated strategies to identify biologically aggressive precancerous breast lesions. Our goal is to identify spatiotemporal changes that occur prior to development of interval breast cancers. To accomplish this requires integration of new technology. Our team has the ability to perform single cell in situ transcriptional profiling, noncontrast biological imaging, mathematical analysis, and nanoscale evaluation of receptor organization and signaling. These technological innovations allow us to start to identify multidimensional spatial and temporal relationships that drive the transition from biologically aggressive precancer to biologically aggressive interval breast cancer.

This article is categorized under:

Cancer > Computational Models

Cancer > Molecular and Cellular Physiology

Cancer > Genetics/Genomics/Epigenetics

## Graphical Abstract

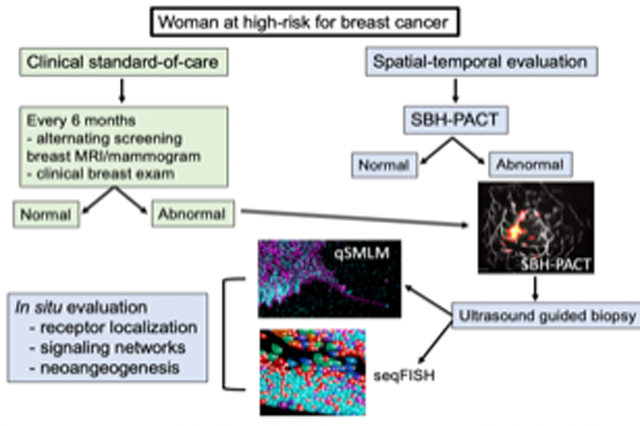

## 1 | INTRODUCTION

The precursor lesion(s) for biologically aggressive breast cancers are poorly understood. Currently, the clinical aggressiveness of a precancerous breast lesion is primarily evaluated based on epithelial cell morphology (normal < hyperplasia < atypia < ductal carcinoma in situ [DCIS] < invasive cancer). While morphology is important, precancerous lesions are clinically assessed at a single time point and on morphology alone, which may not always account for the future biological potential of a precancerous lesion. For example, a 1.0 cm, node-negative, estrogen receptor/progesterone receptor-positive (ER/PR+) HER2-wild type lesion in an 80-year-old woman is treated more aggressively than atypical ductal hyperplasia in a 30-year-old germline *BRCA1* mutation carrier. Yet, the 30-year-old *BRCA1* mutation carrier (relative to the 80-year-old woman) has a much higher 5-year probability of developing metastatic breast cancer.

Currently there is a need to re-think our approach to evaluating precancerous breast lesions and screening for biologically aggressive breast cancer. Despite nationwide mammography screening programs to decrease mortality from breast cancer by increasing early detection, it is not possible to identify which patients diagnosed with atypical biopsies will progress to develop breast cancer. Both the genetic basis for cancer and the multi-hit hypothesis are well supported, but it remains unclear how individual genetic alterations affect the tissue ecosystem, how spatial microenvironments are organized with heterogeneous populations of cell types, and how the evolution of the molecular signatures in these cells in precancerous lesions drive tumor initiation. Understanding the genetic, epigenetic, and transcriptional basis of this transition within the native spatial context is critical to stratify patients for the development of personalized medicine approaches to pair patients with the most appropriate interventional strategies based on their specific prognosis.

## 2 | CURRENT CHALLENGES

### 2.1 | Standard of care guidelines do not adequately account for biology

Breast cancer is a diverse collection of diseases with distinct biology, rate of progression, and prognosis. Despite the heterogeneity of breast cancer as a disease, our strategies for screening and early detection are not tailored to the likely biology of the cancer in question. For example, current National Comprehensive Cancer Network guidelines recommend the same imaging plans for women who harbor a germline *BRCA2*- versus *BRCA1*-mutation (yearly screening mammography and breast magnetic resonance imaging) (Bevers et al., 2018). These uniform imaging plans are recommended despite the fact that women with germline *BRCA2* mutations typically develop average prognosis estrogen receptor-positive (ER+) breast cancer and women with *BRCA1* mutations most frequently develop biologically aggressive triple-negative breast cancers (TNBC) (Ha et al., 2017; Joosse, 2012). Ultimately, our screening approach is successful at detecting biologically indolent disease but frequently fails at early detection of biologically aggressive cancers.

### 2.2 | Precancerous breast lesions—over reliance on DCIS

DCIS has been extensively studied as a precursor lesion. DCIS is easy to identify on mammography, has a long leadtime, and has defined morphology. However, it is doubtful that DCIS is representative of aggressive biology. Even when DCIS progresses to invasion, the resulting invasive breast cancer typically carries a good prognosis (20 year survival is 97%) (Narod, Iqbal, Giannakeas, Sopik, & Sun, 2015). Furthermore, a DCIS intermediate is rarely (<15%) identified for the majority of biologically aggressive cancers (Bryan, Schnitt, & Collins, 2006). It is hypothesized that biologically aggressive breast cancers progress so rapidly as to preclude capture of a DCIS precursor lesion.

### 2.3 | Spatial progression—does breast cancer evolve from a focal mass or high-risk field?

In 1996, Helene Smith first proposed the concept of a “high risk field”—that breast cancer arose in a damaged lobule (or lobules) containing molecular changes that allowed the survival of a “second-hit” (Deng, Lu, Zlotnikov, Thor, & Smith, 1996). It is not clear, even today, whether breast cancer arises from a focal mass (conventional model) or a biochemically damaged breast lobule. If aggressive breast cancers arise from a focal mass, then the best strategy for early detection is high resolution imaging. Conversely, if aggressive breast cancers arise from a high-risk field, then the best approach is to develop biologically based imaging to detect the damaged lobule.

### 2.4 | Contribution of tissue inflammation and need for metabolic imaging

While the association between obesity and postmenopausal, estrogen receptor-positive (ER+) breast cancer is well established, the association between obesity, inflammation, and pre-menopausal breast cancer remains controversial, particularly in Black/African American women (Yee, Mortimer, Natarajan, Dietze, & Seewaldt, 2020). One potential confounding factor is that body mass index (BMI) and body composition (muscle to adipose tissue ratio) differs between race and ethnicity (Gomez-Ambrosi et al., 2012; Okorodudu et al., 2010). It is known that elevated adipose tissue is associated with (a) inflammation of breast white adipose and (b) is associated with adipocyte hypertrophy and cell death leading to chronic, subclinical inflammation of adipose tissue (Iyengar et al., 2019). There has also been a focus on metabolic health; individuals with elevated BMI can be metabolically healthy; conversely individuals with a low or normal BMI can be metabolically unhealthy and insulin-resistant (Iyengar et al., 2017). Insulin activates AKT/mTORas well as Ras/Raf/cMYC-signaling—pathways that are involved in predicting aggressive breast cancer biology (Yee et al., 2020). These studies highlight the importance of being able to image the metabolic state of at-risk and cancerous breast tissue.

### 2.5 | Importance of immune cell signaling and immunotherapy

The last 5 years have brought an increased appreciation of the role of immune cells in cancer initiation and response to targeted immune therapy. While there has been significant progress in developing targeted immunotherapy for many cancer types, progress in breast cancer has been limited. Breast cancers are considered to be immunologically “cold” (quiescent) and exhibit (a) low lymphocyte infiltration and (b) poor response to anti-PD-1/PD-L1 therapy (Gatti-Mays et al., 2019). Tumor and immunologic profiling of the breast tumor microenvironment has identified key immune components and potential mechanisms that immune-cell subset promote evasion to immune therapy. Therapeutic strategies to increase breast cancer response to immunotherapy are reviewed by Gatti-Mays et al. and include (a) expand effector T-cells, natural killer cells, and immunostimulatory dendritic cells, (b) increase the effectiveness of antigen presentation, and (c) decrease inhibitory cytokines, M2 tumor-associated macrophages, and myeloid derived suppressor cells (Gatti-Mays et al., 2019). While all three strategies hold promise, there is a need to be able to rapidly and in real-time (a) characterize the in situ signaling networks that promote a “cold” microenvironment in the breast and (b) determine whether strategies to “warm” the “cold” microenvironment have been successful. Our goal is to develop strategies that can identify in situ immune cell signaling networks and provide real-time metabolic imaging of tissue inflammation and response to targeted prevention and therapy.

## 3 | CANCER INITIATION

### 3.1 | What is the time scale for initiation and progression of aggressive breast cancers?

**FIGURE 1:**
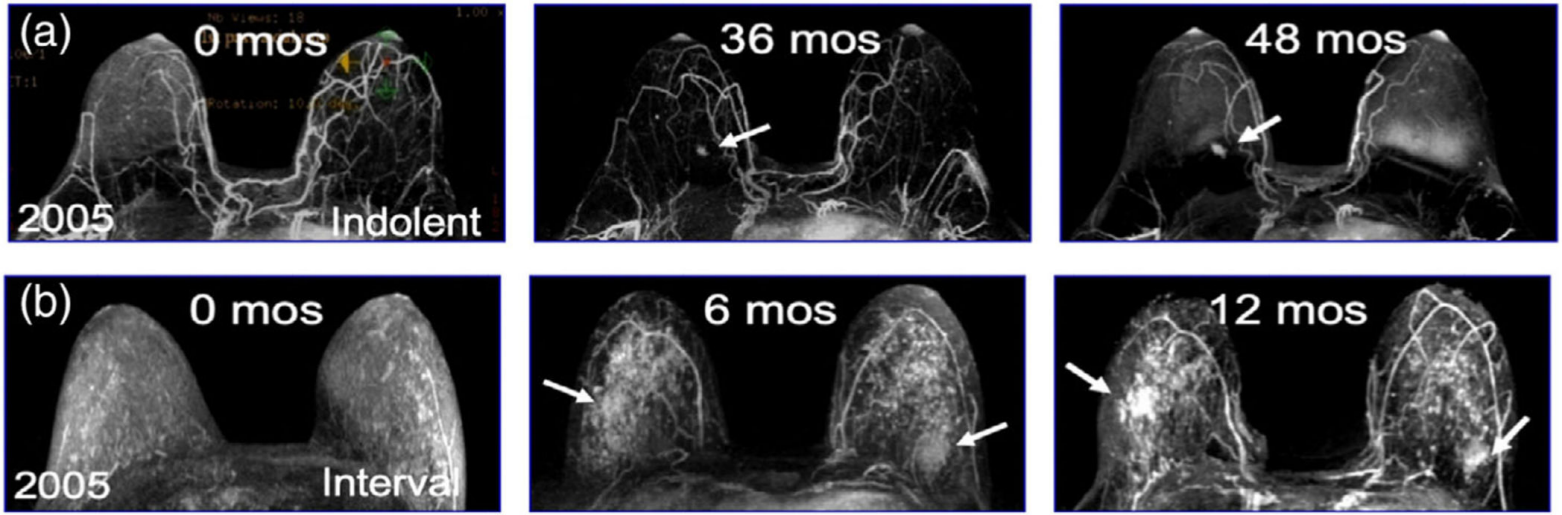
Screening breast MRI imaging of slowly progressing (a) versus rapidly progressing interval (b) breast cancer. (a) Slowly progressing ER/PR+ HER2-wild-type breast cancer; arrow at 36 months indicates when breast cancer was first observed, however at that time, it was too small for our clinical team to biopsy; arrow at 48 months demonstrates slow growth of the tumor over the 12 month interval; the breast cancer was biopsied at that point, staging revealed a T1N0M0 indolent breast cancer (b) Rapidly progressing ER/PR– HER2-wildtype triple negative breast cancer (TNBC), 0 months is a normal MRI, at 6 months a change in MRI patterning was observed, this was one of our first bilateral screening MRI and our team did not recognize the importance of this change and recommended a 6 month follow-up, biopsy at 12 months (see arrows) demonstrated a T2N0M0 TNBC, the woman underwent bilateral mastectomy and chemotherapy, she is currently alive and disease free

Currently, we have a poor understanding of the timescale over which biologically aggressive breast cancers evolve. From 2005 to 2014 we enrolled 656 underserved high-risk women in a disparities breast MRI screening trial. All women had greater than 20% lifetime risk for breast cancer and clinically qualified for breast MRI screening by American Cancer Society Guidelines (Saslow et al., 2007). The racial distribution of these 656 women was: 347 (53%) White, 292 (44%) Black, 14 (2.1%) Asian, 3 (<1%) Native American/Pacific Islander; the ethnic distribution was 98% (648%) non-Hispanic and 8 (2%) Hispanic. From 2005 to 2014, 41 women developed invasive breast cancer; the stage of presentation was 28 (68%) Stage 1, 13 (32%) Stage 2–3, and 0 (0%) Stage 4. Our serial MRI screening studies provide evidence that while some breast cancers progressed slowly over time (Figure 1a), other breast cancers rapidly progressed and metastasized (Figure 1b).

### 3.2 | How early can aggressive biology be detected?

**FIGURE 2:**
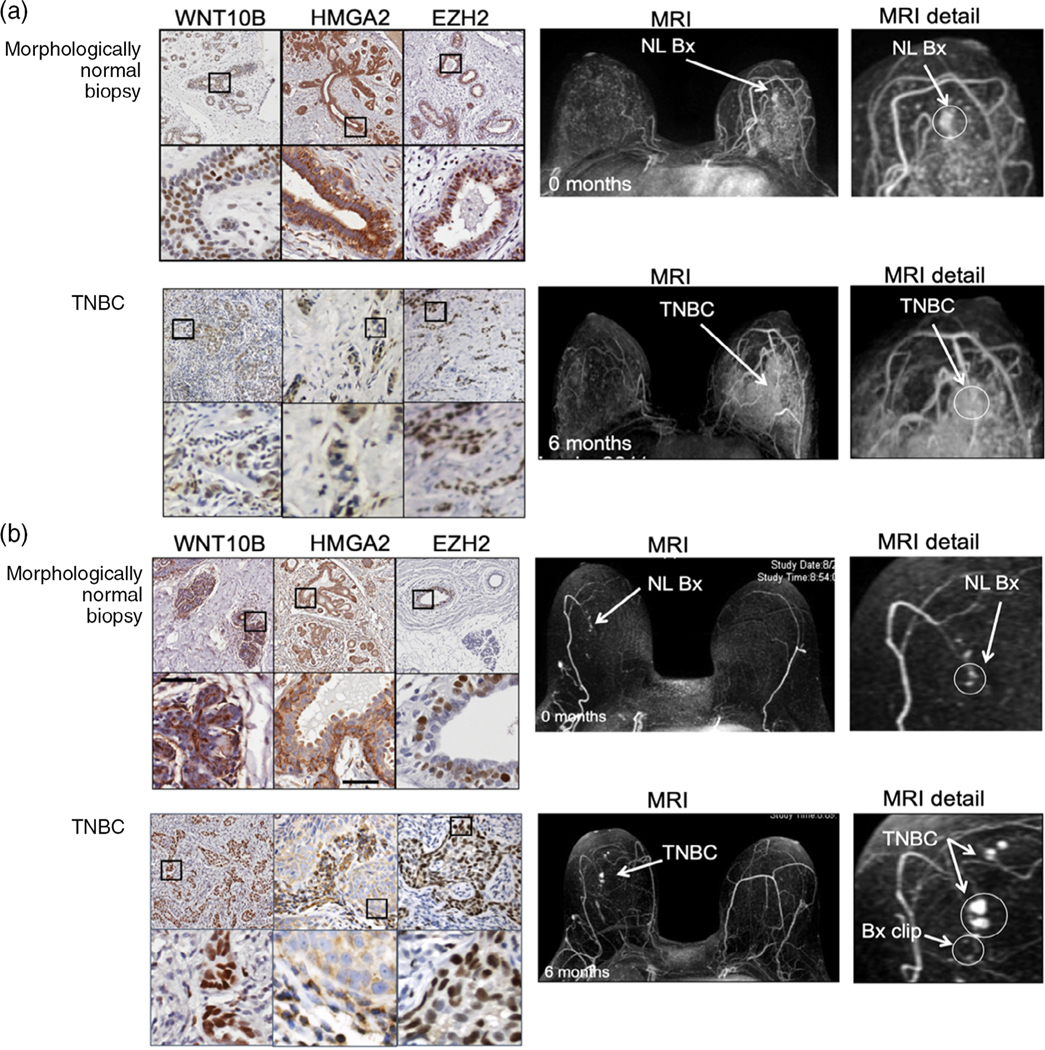
Matched MRI guided biopsy and MRI imaging demonstrates Wnt10B, HMGA2, and EZH2 expression in both morphologically normal biopsy at 0 months and follow up MRI imaging at 6 months. (a) 36-Year-old high-risk premenopausal woman and (b) 61-year-old high-risk woman had an abnormal screening MRI at 0 months and 6 months MRI follow-up. For both women, biopsy at 0 months demonstrated morphologically normal tissue that had high expression of Wnt10B/HMGA2/EZH2; 6 month follow-up biopsy demonstrated TNBC that also expressed high levels of Wnt10B/HMGA2/EZH2, both women had multiple lymph nodes that contained invasive TNBC but no metastatic disease. After greater than 5 years follow-up both women are alive and disease free

Interval breast cancers are cancers that develop within the recommended screening interval. Of the 656 high-risk women participating in our MRI screening trial, 41 women developed an invasive interval breast cancer; of these women, 6 had an abnormal MRI but a normal follow-up biopsy within 6 months of developing an interval breast cancer. These individuals provide an opportunity to retrospectively evaluate signaling proteins expressed in the breast biopsy that preceded the development of an interval cancer. Retrospective analysis of the six paired interval cancer and preceding morphologically normal breast tissue demonstrated, by immunohistochemistry (IHC), increased expression of Wnt signaling proteins including Wnt10B, HMGA2 (High Mobility Group AT-Hook 2), and EZH2 (Enhancer of Zest2) (Figure 2).

Wnt proteins are a family of signaling molecules involved in normal embryologic development and dysregulated in many biologically aggressive cancers, including breast, lung, and colon cancer (Wend et al., 2013). Gene profiling shows that Wnt is frequently activated in TNBC, particularly in the highly aggressive basal-like and mesenchymal-like subtypes (El Ayachi et al., 2019; Wend et al., 2013). Wnt10B-signaling is activated in >90% of TNBC and Wnt10B-activation (unlike Wnt1, Wnt7) predicts poor survival in women with TNBC. We have previously shown that Wnt10B/beta-catenin transcriptionally activates HMGA2. Wnt10B and HMGA2 co-activate EZH2 and activated HMGA2/EZH2 drives a positive feedback loop that further increases EZH2 expression (El Ayachi et al., 2019). Previous studies show that EZH2 regulates stem-cell maintenance, cancer initiation, and chemotherapy resistance (Kleer et al., 2003).

Our study is limited by a small number of interval cancers (n = 6); currently a multi-center prospective study is underway to evaluate the biology of interval cancers. Despite its limitations, our study provides evidence that morphologically normal breast tissue can express proteins such as Wnt10B/EZH2 that are associated with aggressive breast cancer biology.

### 3.3 | Current challenges

Our breast MRI studies provide evidence that some breast cancers progress slowly, but others progress rapidly and metastasize. In limited studies, we observe by IHC that rapid progression of interval cancers is preceded by expression of Wnt-signaling proteins in morphologically normal tissue. This raises the question of whether, in rapidly progressing breast cancers, biochemical abnormalities precede the development of morphological changes (e.g., atypia). To answer this question, we are currently enrolling high-risk women in a prospective MRI screening trial in high-risk women that aims to evaluate the frequency of Wnt-activation in morphologically normal tissue prior to development of an interval cancer. In developing this trial, it was clear that we needed to incorporate analytic tools with the ability to evaluate the precancerous microenvironment. We anticipate that in incorporating new analytic tools for breast cancer early detection we will offer strategies for early detection of additional tumor types. Key questions:

1. What are the spatiotemporal cell–cell interactions (epithelial, stromal, immune cells) in the precancerous microenvironment that drive biologically aggressive breast cancers?
2. Can the spatial and temporal organization of cell surface receptors identify aggressive biology and predict chemotherapy resistance?
3. Can early biology be imaged with high-resolution but without contrast or radiotracers?

## 4 | SINGLE-CELL APPROACHES TO IDENTIFY AGGRESSIVE BIOLOGY

Loss of normal tissue organization is a hallmark of invasive breast cancer and one of the earliest changes observed during breast cancer initiation. In the mammary gland, epithelial cells organize within the epithelial compartment and orient relative to the basement membrane. Stromal cells organize in the stromal compartment with the basement membrane serving as the dividing line. It is also recognized that interactions between immune cells and epithelial/stromal cells play an important role in cancer initiation, progression, and response to therapy. Currently very little is known about the role of immune cell signaling/inflammation in promoting breast cancer initiation.

The dynamic interplay between epithelial, stromal, and immune cells governs normal mammary gland architecture. Loss of these normal regulatory cell–cell signals is hypothesized to play a key role in cancer initiation and progression. Currently there are many approaches developed, or in development, to evaluate gene expression and chromatin expression in single cells—both to understand cancer initiation and progression of aggressive cancer biology.

### 4.1 | Single-cell transcriptome analysis

First reported in 2009, single-cell RNA sequencing (scRNAseq) provides in-depth transcript analysis of single isolated cells. scRNAseq has rapidly revolutionized understanding of temporal processes such as cell differentiation and malignant transformation (Suva & Tirosh, 2019). Key to scRNAseq’s power for in depth analysis is its adaptability; scRNAseq has been adapted for variant detection, DNA methylation, chromatin structure, and multi-omic analysis (Stark, Grzelak, & Hadfield, 2019).

The rapid adoption and success of scRNAseq are also due to the development of user-friendly computational tools for analyzing single cell data. These computational tools allow the user to quantify and interpret expression for each captured cell (Stark et al., 2019). Methods to analyze transcript expression include: (a) dimensionality reduction for visually representing the transcriptome of thousands of cells in two to three dimensions (tSNE and UMAP) (McInnes, Healy, & Melville, 2018; van der Maaten & Hinton, 2008), (b) supervised and unsupervised clustering methods and marker gene identification for identifying transcriptionally similar cell types (TSCAN, SIMLR, MetaNeighbor) (Crow, Paul, Ballouz, Huang, & Gillis, 2018; Ji & Ji, 2016; B. Wang et al., 2018), and (c) pseudotime trajectories for ordering cells based on biological processes (Monocle, PAGA) (Trapnell et al., 2014; Wolf et al., 2019).

Single cell analysis has great power to evaluate the temporal evolution of cell lineage and tissue heterogeneity in both normal and malignant tissue. However, current techniques are limited because, to perform the analysis, tissue must first be dispersed into single cells. In dispersing tissue into single cells, spatial organization of the microenvironment (spatial environment) is lost. The spatial environment consists of cell–cell interactions, organization, and signaling. These interactions play a vital role in cancer initiation and progression. Consequently, new in situ techniques have recently been developed to evaluate the transcriptome of single cells without disrupting tissue structure and organization, particularly in the study of tissue inflammation, immune cell-signaling, and cancer initiation and progression.

### 4.2 | In situ single cell profiling

Recently, several approaches have been developed to provide spatial analysis of gene expression and chromatin structure in single cells. These single cell spatial approaches are reviewed below. Each technique holds promise, but is limited either by low resolution or lack of commercialization. It is anticipated, in the near future, that these approaches will grow in their sophistication and become more accessible to investigators.

### 4.3 | In situ scRNAseq

This technique provides spatial analysis by physical transfer of nuclear material from individual cells within a tissue slice to a slide for analysis (Rodriques et al., 2019; Salmén et al., 2018; Stahl et al., 2016). To perform analysis, a tissue slice is applied to a slide coated with oligo spot or DNA barcoded beads, RNA or DNA is released and analyzed relative to the spatial location of the original cell. This in situ single cell analysis tool, Spatial Transcriptomics, is commercially available from ×10 Genomics and has been tested in breast cancer biopsy specimens (Stahl et al., 2016). The limitations of Spatial Transcriptomics are that (a) tissue sections cannot exceed 7 mm^2^ in size and (b) the RNA capture spots (100 μm diameter) significantly exceed the diameter of a single cell (10 μm diameter). A second in situ method proposed by Rodrigues et al., called Slide-seq, provides significantly improved spatial resolution (10 μm) (Rodriques et al., 2019). A third approach, Geo-seq uses spatially targeted laser capture microdissection (LCM) (J. Chen et al., 2017). The disadvantages of Geo-seq are that the protocol is time consuming and requires access to a spatially targeted LCM system (J. Chen et al., 2017; Stark et al., 2019).

### 4.4 | seqFISH

**FIGURE 3:**
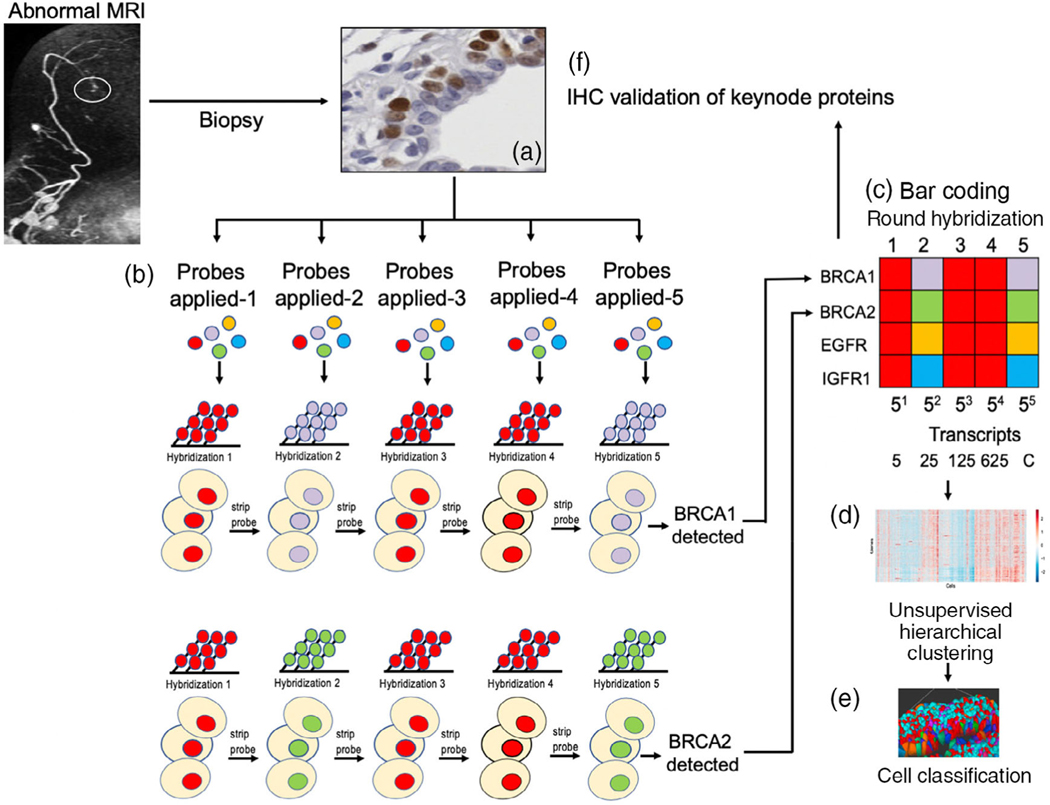
Schema depicting the use of seqFISH for evaluating transcript expression in biopsy tissue. (a) Biopsy tissue is obtained and preserved. (b) Tissue undergoes successive rounds of mRNA hybridization and probe stripping. (c) Transcripts are identified by specific bar code identification (BRCA1 and BRCA2 are used as illustrations. (d) Transcript expression is analyzed for each transcript in each cell using unsupervised hierarchical clustering. (e) Cells are classified based on expression pattern, a color assigned for each cell type, and the tissue architecture is reconstructed. (f) Keynode proteins are validated by IHC

Single molecule fluorescence in situ hybridization (smFISH)^28,29^ has been used for over 15 years to visualize and quantify RNA and DNA in single cells, in situ, in tissue sections. However, smFISH is insufficient for transcript profiling due to a limited number of fluorophores and channels. First reported in 2012, Dr Long Cai’s lab has developed a general method to highly multiplex single molecule in situ mRNA imaging called sequential FISH (seqFISH) (Figure 3) and has the capacity to profile hundreds of genes in situ (Lubeck & Cai, 2012; Lubeck, Coskun, Zhiyentayev, Ahmad, & Cai, 2014). Building upon smFISH, seqFISH uses a temporal barcoding scheme that relies on a limited set of fluorophores and scales exponentially with time. Specifically, sequential probe hybridizations on the mRNAs in fixed cells impart a unique predefined temporal sequence of colors, generating in situ mRNA barcodes. The multiplex capacity scales as F^N^, where F is the number of fluorophores and N is the number of rounds of hybridization. Thus, one can increase the multiplex capacity by increasing the number of rounds of hybridization with a limited pool of fluorophores. With 4 fluorophores, 256 genes can be multiplexed with 4 rounds of hybridization.

### 4.5 | Use of seqFISH to identify aggressive biology in precancerous breast tissue

Currently, Dr Long Cai is applying seqFISH for the first time in a cancer-related setting. One key advantage of seqFISH is that it enables multi-modal data collection in the same sample with the native spatial structure of the tissue preserved without disruption or dissociation. In addition to mRNA, seqFISH^30^ can also simultaneously evaluate introns in single cells in situ (intron seqFISH). Introns often appear only near transcription active sites and are rapidly degraded posttranscription. Thus, the short-lived nature of intron measurements informs the instantaneous transcriptional program that the cells are executing. Recent work, called RNA velocity (La Manno et al., 2018), showed that the intron to exon ratio can predict the differentiation directions of the cell. Techniques like seqFISH provide power to evaluate changes in the precancerous microenvironment. seqFISH has the ability to simultaneously evaluate early changes in cell–cell paracrine signaling and evaluate the spatiotemporal dynamics of the earliest changes in cancer initiation—potentially even before substantial morphological changes occur.

## 5 | NANOSCALE CELL SURFACE RECEPTOR ORGANIZATION

### 5.1 | Super resolution microscopy

The wavelength of visible light (~250 nm) was once thought to be an insurmountable limit for microscopy. This barrier has been surpassed by the development of super resolution microscopy (SRM) technologies. SRM now provides <1 nm resolution of cellular organization and can be used to investigate receptor signaling. SRM, in combination with fluorescent tags, allows for measurement of spatiotemporal dynamics of specific DNA and RNA molecules as well as proteins.

Many SRM techniques exist; each SRM technique has specific strengths and weaknesses. Selecting the appropriate SRM technique requires careful consideration of the experimental question being asked. The tradeoffs between the currently available methods involve: (a) lateral and axial resolution limits, (b) depth of image (thickness of sample), (c) time to collect an image, and (d) photodamage (Schermelleh et al., 2019). A number of recent reviews outline the available SRM techniques in terms of their strengths and limitations and can help guide researchers in the selection of a SRM technique (Sage et al., 2019; Schermelleh et al., 2019; Sigal, Zhou, & Zhuang, 2018).

### 5.2 | Predicting aggressive biology and chemotherapy-resistance in a real-time clinical setting

Single molecule localization microscopy (SMLM) is an SRM approach in which blinking fluorescent molecules are localized with nanoscale precision. Dr Tijana Jovanovic-Talisman has pioneered the development and application of quantitative SMLM (qSMLM) for investigation of receptors in patient tissue samples (Jorand et al., 2016; Tobin et al., 2018).

**FIGURE 4:**
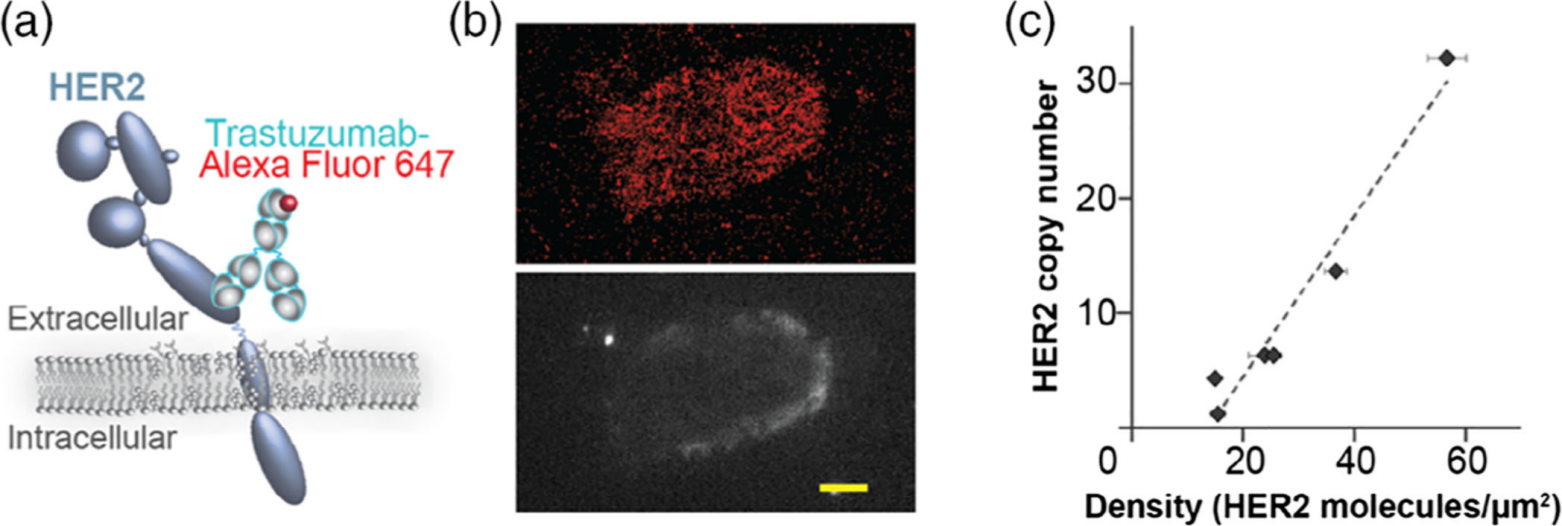
Touch prep-qSMLM for imaging of HER2 in breast cancer tissues. (a) Scheme of HER2 receptor detected using fluorescently labeled trastuzumab. Alexa Fluor 647 was the fluorophore used in this case. (b) Image of HER2 positive breast cancer patient cell. Top, red signal represents localizations of Alexa Fluor 647 labeled trastuzumab detected with dSTORM. Bottom, gray signal represents signal from anti-cytokeratin 7 antibody detected with diffraction limited microscopy. Scale bar, 5 μm. (c) Average detected HER2 density obtained with touch prep-qSMLM versus HER2 copy number obtained from FISH. For six patients, the correlation coefficient was 0.979 with 95% CI [0.813, 0.998] with a *p*-value of .0007. Image adapted from Tobin et al. (2018)

We detected HER2 in patient samples with fluorescently labeled trastuzumab (Figure 4a). This therapeutic antibody allowed us to directly detect the HER2 extracellular domain. Labeled samples were imaged using direct optical stochastic reconstruction microscopy (dSTORM) with an optimized acquisition protocol (Tobin et al., 2018). An example dSTORM image of HER2 positive cell is shown in Figure 4b (top) a diffraction limited image of the same cell stained with antibody against epithelial marker, cytokeratin 7, is shown in Figure 4b (bottom). The qSMLM readout obtained on the extracellular HER2 domain may be clinically relevant. Trastuzumab does not bind to constitutively active variant of HER2 with truncated extracellular domain; patients harboring this variant have worse outcome (Saez et al., 2006; Scaltriti et al., 2007). Other poor responders have a diminished pool of the sterically accessible extracellular HER2 epitope (Mercogliano et al., 2017).

Quantification of qSMLM provides real-time information on receptor density and clustering across the membranes. Additionally, we can assess receptor heterogeneity. Importantly, we have shown that HER2 copy numbers from FISH had a significant positive correlation with detected densities from touch prep-qSMLM (Tobin et al., 2018) (Figure 4c).

### 5.3 | Real-time evaluation of aggressive biology

The application of qSMLM to cancer research has promise to increase our understanding of receptor signaling and localization during cancer initiation. Increasing evidence suggests that receptor localization may play a key role in defining aggressive biology and chemotherapy resistance. The advantage of qSMLM is that it can provide real-time evaluation (6 hr) of receptor biology in a small clinical sample (touch-prep).

Currently we are using qSMLM to evaluate aggressive biology in luminal B breast cancers (ER+/HER2-wt or -amplified, Ki67 > 14%). The majority of luminal B breast cancers carry a good prognosis. However, there is a subset of luminal B breast cancers that are biologically aggressive and highly lethal. These aggressive luminal B breast cancers disproportionately impact young Black/African American and Hispanic/Latina women of African ancestry (Jemal, Center, DeSantis, & Ward, 2010; Ooi, Martinez, & Li, 2011; Serrano-Gomez et al., 2016, 2017).

In luminal B breast cancer, membrane receptors such as HER2, HER3, and EGFR (HER1) can reside and interact within cholesterol enriched domains, thus creating activated receptor platforms that support rapid signaling (Gueguinou et al., 2015; Marquez & Pietras, 2001). Upon ligand binding, these activated receptors phosphorylate RAS/ERK/AKT kinases (Ades et al., 2014; Gueguinou et al., 2015; Marquez & Pietras, 2001; Pietras et al., 1995) and can upregulate c-MYC (Z. Chen, Wang, Warden, & Chen, 2015; Kerkhoff et al., 1998; R. Sears et al., 2000; R. C. Sears, 2004). Studies of luminal B breast cancers have identified two key drivers of aggressive biology in women of African ancestry: (a) c-myc (MYC) (Naab et al., 2018) and (b) HER2/pAKT-coactivation (Y. Wu et al., 2008). We are currently using touch prep-qSMLM to assess the role of IGF1R-HER2 interaction in driving aggressive luminal B biology. qSMLM is being used in a research setting in Black and Latina women with luminal B breast cancer to define HER2/IGF1R density and colocalization as a means to drive drug development and new therapeutic approaches.

## 6 | REAL-TIME HUMAN NONCONTRAST BIOLOGICAL IMAGING

### 6.1 | Limitations of current imaging for early detection

Breast MRI has significantly improved high-risk breast cancer screening. However, MRI has significant limitations. MRI is expensive and requires the use of gadolinium intravenous contrast agents that can cause hepatorenal syndrome (Perazella, 2008), permanent deposition in the brain (Ibrahim, Froberg, Wolf, & Rusyniak, 2006), and anaphylaxis (Ramalho & Ramalho, 2017). Furthermore, while MRI is highly sensitive, MRI has limited specificity. Due to high sensitivity and low specificity, many women undergoing high-risk breast MRI screening require follow-up MRI imaging, second-look ultrasound, and biopsy. The vast majority of MRI-generated biopsies do not identify cancer, but still require expensive follow-up. The follow-up is required because while we can determine that (a) an MRI is “abnormal” and (b) the resultant MRI-generated biopsy is “not cancer,” we are unable to determine the future biological potential of the “not cancerous” biopsy. Our study of high-risk women previously described above (Figure 2) provides evidence that some MRI-detected non-cancerous biopsies demonstrate activation of signaling pathways that predict aggressive biology in invasive cancer. There is a need for new imaging techniques that can rapidly and reproducibly image early aggressive cancer biology without the need for intravenous contrast agents.

### 6.2 | Optical imaging and photoacoustic computed tomography

Optical imaging uses light (photons) to activate endogenous molecules (e.g., NADH and hemoglobin) within living cells and tissues. No contrast injection is required. Optical imaging holds great promise for early detection of aggressive biology. Until recently, high-resolution optical imaging could not be used for breast imaging because the penetration is limited to 1–2 mm.

Photoacoustic computed tomography (PACT) is a novel noncontrast imaging technique that combines endogenous optical contrast with ultrasonic spatial resolution in a single imaging system (Razansky et al., 2009; Xia, Yao, & Wang, 2014). PACT holds promise to provide high-resolution optical (endogenous) imaging of deep tissues. Several PACT systems have been developed that employ a variety of light illumination and detection schemes (Ermilov et al., 2009; Fakhrejahani et al., 2015; Heijblom, Piras, et al., 2015; Ke, Erpelding, Jankovic, Liu, & Wang, 2012; Kitai et al., 2014; Kruger et al., 2013; X. Li, Heldermon, Yao, Xi, & Jiang, 2015; Toi et al., 2017; D. Wang et al., 2017). These systems have advanced PACT technology, but clinical implementation has been blocked by a number of technical challenges. These technical challenges include: (a) insufficient penetration depth to accommodate most breast sizes and skin colors, (b) inadequate spatial resolution to image detailed vascular patterns, (c) lack of high temporal resolution to minimize motion artifacts and enable dynamic or functional studies, (d) minimal limited-view artifacts, and (d) insufficient noise-equivalent sensitivity and contrast-to-noise ratio to detect breast masses (Ermilov et al., 2009; Fakhrejahani et al., 2015; Heijblom, Piras, et al., 2015; Heijblom, Steenbergen, & Manohar, 2015; Ke et al., 2012; Kitai et al., 2014; Kruger et al., 2013; Toi et al., 2017; D. Wang et al., 2017).

### 6.3 | Recent advancements in PACT imaging

Dr Lihong Wang and associates, as pioneers in the development and translation of PACT for breast imaging (Yao, Xia, & Wang, 2016), recently reported a significant advancement in breast PACT technology, single breath-hold PACT (SBHPACT) (Lin et al., 2018). SBHPACT overcomes the five technical challenges listed above that have impeded implementation of PACT imaging. The resolution of SHB-PACT is approximately four times greater than the resolution of contrast-enhanced MRI (Lehman & Schnall, 2005). SBHPACT has the capacity to obtain (a) an entire 2D cross-sectional breast image within 150 μs or (b) a volumetric 3D image of the entire breast within a single breath-hold (15 s). SBHPACT provides dynamic imaging and delivers high image quality (Lin et al., 2018). Capitalizing on the optimized illumination method and signal amplification, SBHPACT achieves sufficient noise-equivalent sensitivity to clearly reveal detailed angiographic structures both inside and outside breast tumors without the use of exogenous contrast agents (Lin et al., 2018).

### 6.4 | SHBPACT breast imaging

**FIGURE 5:**
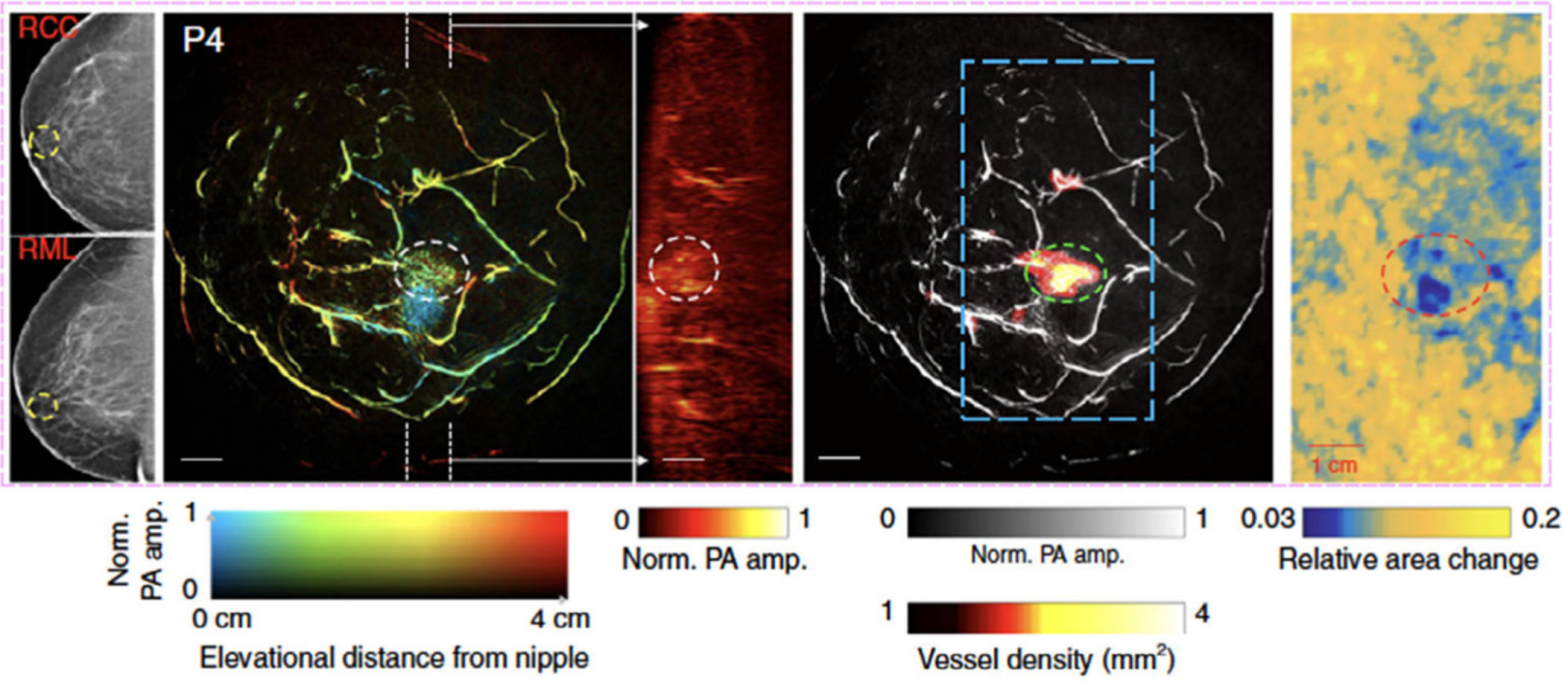
Example of SBHPACT imaging of a cancerous breast. (a) Mammogram of the affected breasts; white circle identified the breast cancer (RCC right cranial-caudal, RML right medio-lateral). (b) Depth-encoded vascular breast imaging acquired by SBHPACT. (c) Maximum amplitude projection (MAP) images of thick slices in sagittal planes marked by white dashed lines in (b). (d) Automatic tumor detection on vessel density map. Background images in gray scale are the MAP of vessels deeper than the nipple. Maps of the relative area change during breathing in the regions outlined by blue dashed boxes in the angiographic images (e) Elastographic study of the affected breast

In published pilot studies, Dr Wang used SBHPACT to image both normal volunteers and women with breast cancer (Yao et al., 2016). In initial studies, SBHPACT identified eight of nine breast tumors by delineation of vascular density and architecture. To improve on the interpretation of images, an algorithm was developed to highlight tumors automatically (Yao et al., 2016). At such high spatiotemporal resolutions, SBHPACT is able to differentiate arteries from veins by detecting blood flow-mediated arterial deformation at the heartbeat frequency. Breast cancers were identified by SBHPACT, even occult cancers that were not identified by mammography (Yao et al., 2016). Taking advantage of the high imaging speed, Dr Wang used elastographic SBHPACT to identify the only (1 of 9) tumor missed by angiographic SHBPACT (Figure 5; adapted from Yao et al., 2016); this innovation improved the sensitivity for breast cancer (Yao et al., 2016).

### 6.5 | Promise of SBHPACT for spatiotemporal imaging

SBHPACT has the power to detect the earliest temporal and spatial biological changes that precede development of biologically aggressive cancers. In our studies of high-risk women undergoing annual breast MRI screening (2005–2014; Figure 2) described above, we observe that clinical neovascularization frequently precedes development of biologically aggressive breast cancer. PACT readily identifies neo-vascularization with substantially higher resolution than breast MRI. Since PACT relies on endogenous signaling (no contrast), PACT can be used to serially track the spatiotemporal events that occur during cancer initiation, particularly, neovascularization. PACT also has promise in detecting tissue inflammation and potentially could be used to monitor immune cell activation and response to immunotherapy.

## 7 | MULTISCALE INTEGRATION OF SPATIOTEMPORAL INFORMATION

### 7.1 | Multiscale integration

In the “big data” age of cancer research, it is increasingly common for multiple data types to be generated for the same patient or patient cohort. Integration of multiple different data types (imaging, −omics, microscopy, clinical data, etc.) for the purpose of addressing a research question is described as multiscale integration. Multiscale integration is motivated by the fact that cancer is ultimately one process in a complex biological system. Cancer evolves over both space and time, and the effects of cancer span from atomic-scale interactions to macro-scale organization of tissues. Therefore, to investigate the system wide effects of cancer, multiple data types that cover the scope of a cancer’s effects are needed. Disparate data types provide unique and complementary information as they are obtained from different technologies, experimental techniques, time points, and spatial scales (micro- vs. macro-scale). Data types that are more complementary to each other contain more independent information; however, the tradeoff is that data types that are more independent are often more challenging to develop successful integration strategies.

### 7.2 | Defining multiscale integration strategies

Multiscale integration seeks to extract the independent information from each data type that is relevant to the research question. This task is non-trivial as the data types involved can rarely be quantified in a way that allows direct harmonization. Mathematical and information theoretic approaches are helpful in determining how two data types are dependent (Ritchie, Holzinger, Li, Pendergrass, & Kim, 2015). Integration is typically performed by identifying or structuring the data with respect to an outcome or an explanatory variable. Computer learning algorithms have emerged as a promising avenue as a general multiscale integration framework (Y. Li, Wu, & Ngom, 2018). Artificial intelligence algorithms are a good fit for data integration because they can agnostically select the aspects of the data that are most informative to a particular research question.

## 8 | CONCLUSION

**FIGURE 6:**
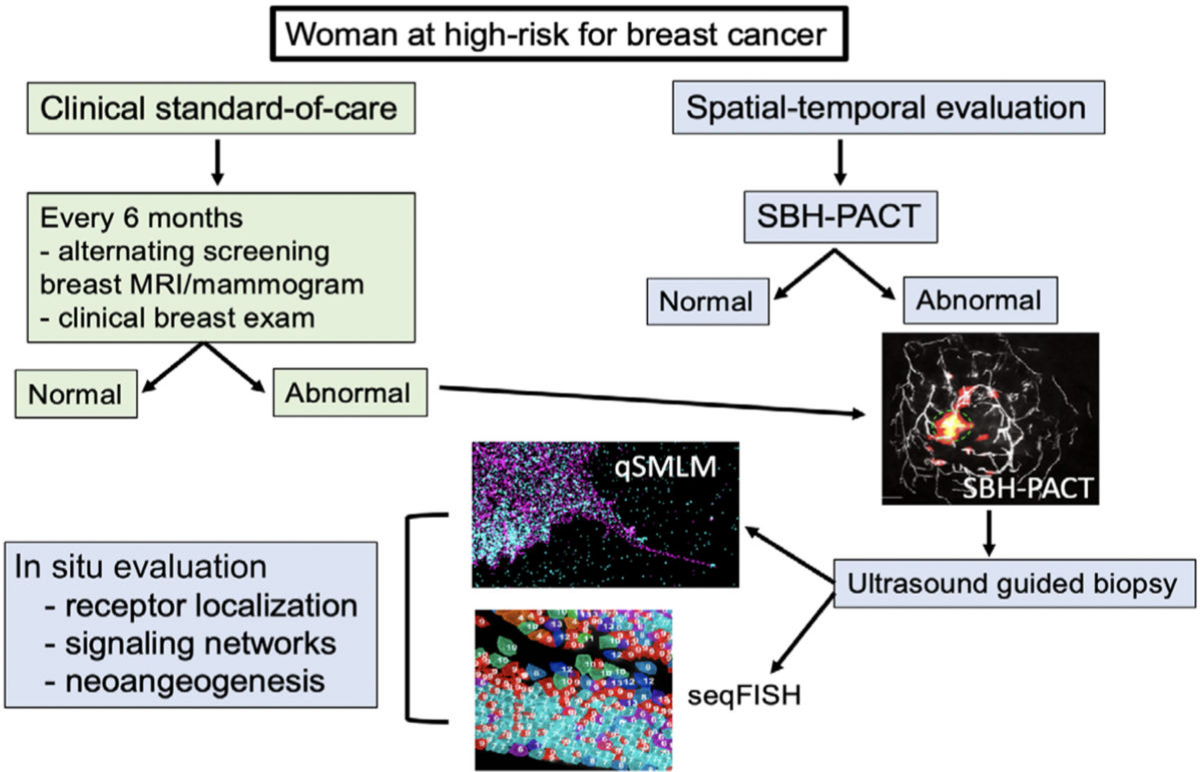
Current multi-modality schema for evaluating aggressive biology in precancerous and cancerous breast tissue. qSMLM, quantitative-single molecule localization microscopy; SBHPACT, single breath-holding photoacoustic computed tomography; seqFISH, sequential fluorescence in situ hybridization

Currently, we lack integrated strategies to identify spatiotemporal events surrounding cancer initiation and progression. With the evolution of targeted immunotherapy, there is the need for real-time tools to monitor immune microenvironment activation. We anticipate that the addition of new analytic tools such as seqFISH, qSMLM, and SBHPACT will provide us with the ability to evaluate early events in the precancerous and malignant microenvironment (Figure 6). Unlike breast MRI, SBHPACT can be repeated multiple times over a span of hours, allowing for real-time evaluation of metabolism (e.g., following oral glucose administration), and rapid assessment of response to prevention agents and agents that modify immune cell activation. It is our hope that integration of these new tools can provide a roadmap for early identification of aggressive biology, not just in breast cancer but for other tumor types.
